# Predatory Ability of *Harmonia axyridis* (Coleoptera: Coccinellidae) and *Orius sauteri* (Hemiptera: Anthocoridae) for Suppression of Fall Armyworm *Spodoptera frugiperda* (Lepidoptera: Noctuidae)

**DOI:** 10.3390/insects12121063

**Published:** 2021-11-26

**Authors:** Ning Di, Kai Zhang, Qingxuan Xu, Fan Zhang, James D. Harwood, Su Wang, Nicolas Desneux

**Affiliations:** 1 Shandong Provincial University Key Laboratory of Protected Horticulture, Weifang University of Science & Technology, Weifang 262700, China; ento88@163.com (N.D.); nicolas.desneux@sophia.Inra.fr (N.D.); 2 Beijing Key Laboratory of Environment Friendly Management on Fruit Diseases and Pests in North China, Institute of Plant Protection, Beijing Academy of Agriculture & Forestry Sciences, Beijing 100097, China; xuqxfarmer@126.com (Q.X.); zf6131@263.Net (F.Z.); jd_harwood@hotmail.com (J.D.H.); 3 Development Center of Science and Technology, Ministry of Agriculture and Rural Affairs, Beijing 100122, China; zhangkai6868@126.com; 4 Institut Sophia Agrobiotech, Université Côte d’Azur, INRAE, CNRS, UMR ISA, 06000 Nice, France

**Keywords:** invasive pest, predator–prey interactions, predatory functional response, biological control

## Abstract

**Simple Summary:**

*Spodoptera frugiperda* (JE Smith) invaded China, dispersed quickly and poses a major threat to agriculture. The predation potential of two species of predators, *Orius sauteri* (Poppius) and *Harmonia axyridis* (Pallas), were tested using functional response experiments in a laboratory. The predation potential of nymphs of *O. sauteri* and adults of *H. axyridis* on eggs and larvae of the pest fitted Holling’s Type II functional response models. Our findings revealed the potential of the two biocontrol agents to control *S. frugiperda*.

**Abstract:**

The lepidopteran pest, *Spodoptera frugiperda* (JE Smith), spread rapidly after its first detection in China and has caused significant yield loss to maize production in the southwestern part of the country. Although natural enemies of *S. frugiperda* are present in the field, biological control using naturally distributed predators is ineffective because their underlying populations are too low. To enhance our understanding of the potential role of natural enemies in regulating this invasive pest, functional response experiments were conducted to quantify the response of two predators, *Orius sauteri* (Poppius) (Hemiptera: Anthocoridae) and *Harmonia axyridis* (Pallas) (Coleoptera: Coccinellidae), in terms of consumption of *S. frugiperda*. Experimental results revealed that the predatory effects of nymphs of *O. sauteri* and *H. axyridis* on the eggs and larvae of *S. frugiperda* fitted Holling’s Type II functional response model. Importantly, the theoretical maximum number of prey consumed per day (*N_a-max_*), the instantaneous attack rate (*a′*) and the handling time (*Th*) of *O. sauteri* nymphs on *S. frugiperda* eggs were 15.19, 0.7444 and 0.049 d, respectively; and the parameters on first instar larvae of *S. frugiperda* were 700.24, 0.5602 and 0.0008 d, respectively. These data contrast to those of *H. axyridis*, where the *N_a-max_*, *a′* and *Th* of adults on eggs of *S. frugiperda* were 130.73, 1.1112 and 0.085 d, respectively, and on the first instar larvae of *S. frugiperda* were 1401.1, 0.8407 and 0.0006 d, respectively. These results revealed that *H. axyridis* is a highly voracious predator of the eggs and young larvae of *S. frugiperda* and *O. sauteri* could also be used as biocontrol agent of this pest. Our work provides a theoretical framework for the application of natural enemies to control *S. frugiperda* in the field. Further research is required to strategize conservation biological control approaches in the field to increase populations of these predators and enhance the suppression of *S. frugiperda*.

## 1. Introduction

The fall armyworm, *Spodoptera frugiperda* (JE Smith) (Lepidoptera: Noctuidae), originated in tropical and subtropical areas of America [1], and its ability for rapid migration and diffusion has resulted in worldwide expansion [2,3]. Presently, this lepidopteran pest occurs in 96 countries or regions, contributing to it becoming a major migratory pest in the global warning system of FAO (Food and Agriculture Organization of the United Nations) [4]. *Spodoptera frugiperda* is a polyphagous pest with over 300 host plants, including maize, cotton, rice, peanut, sorghum, beet, soybean, tobacco, tomato, potato, onion, and wheat (https://www.cabi.org/ISC/datasheet/29810; accessed on 18 November 2021). Such a diverse feeding range poses a major threat to worldwide crop production and was first reported in Southwest China in December 2018, quickly dispersing northward thereafter and now occurs in 385 counties (cities and districts) of 14 provinces, covering an area of 9.23 thousand hectares [5,6].

The rapid spread of *S. frugiperda* throughout China has driven research efforts towards identifying effective control strategies that reduce crop damage. Chemical control has been largely applied [7], but the unsustainable nature and environmental toxicity of pesticides make it unwelcome in modern agriculture, especially in organic production. In addition, coupled with the inefficiency of pesticide application due to the feeding behavior of these pests whereby they feed on the underside of leaves and can bore into plants and fruits, make alternative approaches preferable for long-term management. More importantly, long-term application of pesticides has resulted in resistance to many classes, including carbamates, organophosphates and pyrethroids [8] and multiple side effects on non-target arthropods and human health have been widely documented [9,10]. Given these factors and the negative effects of chemical control [11], there is an urgent need to explore effective sustainable and/or auxiliary chemical control methods to achieve successful pest regulation [12]. Considerable attention has been afforded to the protection, utilization and release of natural enemies for biological control. For example, parasitoids *Glyptapanteles creatonoti* (Viereck) (Hymenoptera: Braconidae) and *Campoletis chlorideae* Uchida (Hymenoptera: Ichneumonidae) were found to attacked the larvae or eggs of *S. frugiperda* in India [13]; *Cotesia icipe* Fernández-Triana & Fiaboe (Hymenoptera, Braconidae) was reported to be the most dominant parasitoid in Africa [14]. However, studies on predators of this pest are relatively rare. Field investigations in maize revealed high populations of *Orius sauteri* (Poppius) (Hemiptera: Anthocoridae), *Chrysopa* *pallens* (Rambur) (Neuroptera: Chrysopidae) and *Sympetrum croceolum* (Selys) (Odonata: Libellulidae), with significant numbers feeding on *S. frugiperda* larvae (pers. obs., Ning Di). The generalist predator, *O. sauteri*, is widely distributed in maize fields and some of these natural enemies acting in concert with each other could provide effective levels of pest control.

Predators belonging to Anthocoridae are useful in biological control programs and have been utilized in field and greenhouse settings [15,16,17,18,19]. *Orius sauteri,* exhibits high predation rates and routinely prey on aphids, leafhoppers, thrips, leaf mites, whiteflies and newly hatched larvae and eggs of Lepidoptera [20]. Importantly, it has attracted widespread attention for biological control in China and plays an important role in many field crops. *Orius sauteri* is particularly common in Northern China and is very abundant in Liaoning (Xingcheng), Beijing, Tianjin, Hebei, Shanxi, Hubei, and Sichuan [21]. The predatory efficiency of natural enemies could be evaluated by functional response models, which are widely used to quantify consumption of natural enemies under prey with different densities, mainly including Holling’s Type I, II and III models [22]. Among the models, the predation of predators in Holling’s Type I is proportional to prey density, and finally reaches saturation; Holling’s Type II, the attack speed of a predator, initially increases with an increase in prey density, and then the rate of increase slows down to saturation; and Holling’s Type III, S-curve, the searching speed of predators is an increase of index with an increase in prey density, but gradually decreases to saturation [23]. When it comes to functional response models of predators on pests, Holling’s Type II models are much more commonly reported than Type III. The latter is often used as a predictor of predators to regulate the population of pests [24]. Previous research revealed the predatory response of *O. sauteri* to *Frankliniella occidentalis* (Pergande) [25] and pseudopupae of *Bemisia tabaci* (Gennadius) [19] were consistent with the Holling’s Type II functional response model. Similar results were reported by Ge et al. (2018) [26] when examining the predatory capacity of *O. sauteri* on *Aphis craccivora* Koch and other research corroborates these patterns on *Orius albidipennis* (Reuter) consumption of *Megalurothrips sjostedti* (Trybom) [27].

*Harmonia axyridis* (Coleoptera: Coccinellidae) is native to Northeastern Asia and is an important natural enemy of many pests, including various hemipterans, such as aphids, mites and scale insects, and the larvae and pupae of Coleoptera, Hymenoptera, Diptera, and Lepidoptera [28,29,30,31]. They are frequently touted as valuable agents in agricultural production throughout the world [32] and the functional response and predatory capacity of *H. axyridis* has been widely studied [33,34,35,36], providing an important baseline of information for the utilization of *H. axyridis* in biological control. In addition to the abundance and predation behavior we observed in the field, *O. sauteri* and *H. axyridis* are among the most commonly used and mass-reared predators, and can be easily purchased by growers. Thus, it is important to evaluate the predation ability of the two kinds of natural enemies on the invasive pest, which helps to develop biocontrol strategies in the field.

There are many approaches to biological control and the protection and utilization of local agents is becoming increasingly important for open field management of insect pests [11,37]. These conservation biological control strategies are often considered optimal due to their relative low cost compared to rear and release methods. It is therefore necessary to evaluate native natural enemies for the management of *S. frugiperda* prior to their utilization in a region. To examine this, we conducted a series of experiments to quantify the predatory behavior and response of two native predators on *S. frugiperda* to evaluate their potential role in biological control. 

## 2. Materials and Methods

### 2.1. Study Organisms

#### 2.1.1. *Spodoptera frugiperda*

Samples of *S. frugiperda* were collected from maize fields from the experimental campus of the Plant Protection and Plant Inspection Station (PPPIS) in Kaiyuan, China (GPS location: 103°27′61″ N, 23°71′75″ E) in May 2019. The insects were transferred to the laboratory at PPPIS and maintained in cages (60 cm × 60 cm × 60 cm), covered with 80-mesh gauze in a temperature-controlled insect feeding room (25 ± 2 °C, relative humidity 60% and photoperiod 16 h:8 h (L:D)) and fed daily with fresh maize leaves.

#### 2.1.2. *Orius sauteri* and *Harmonia axyridis*

*Orius sauteri* and *H. axyridis* were all taken from established colonies at the Laboratory of Applied Entomology, Institute of Plant and Environment Protection, Beijing Academy of Agricultural and Forestry Sciences (BAAFS), Beijing, China. *Orius sauteri* were reared on eggs of *Corcyra cephalonica* (Stainton) (Lepidoptera: Pyralidae) with common bean *Phaseolus vulgaris* L. as the plant substrate. *Harmonia axyridis* were derived from a breeding population in the laboratory that were fed on *Megoura japonica* (Matsumura) (Hemiptera: Aphididae) that were reared on *Vicia faba* L. Both predators were maintained on a photoperiod of 16 h:8 h (L:D) and temperature of 25 ± 2 °C. *Harmonia axyridis* were selected randomly as adults, at 7 and 12 days after emergence, and *O. sauteri* were selected as 5-day nymphs. All predators were starved for 24 h prior to the experiment.

### 2.2. Experimental Methodology

#### 2.2.1. Predatory Capacity of *Orius sauteri* and *Harmonia axyridis* on *Spodoptera frugiperda* Larvae

First instar larvae of *S. frugiperda* were placed in a petri dish (9 cm in diameter) with fresh corn leaves at room temperature and water was provided with moistened cotton balls. The densities of larvae were 10, 20, 40, 60, and 80 per dish and replicated 15 times per treatment. A single *O. sauteri* was placed into each dish and covered with a layer of breathable gauze. After 24 h, the remaining larvae was recorded and a control group was established at the same density (10, 20, 40, 60 and 80 larvae per dish) without any predators and natural mortality rates were recorded. The predatory behavior of the two species was evaluated in the same way.

#### 2.2.2. Predatory Capacity of *Orius sauteri* and *Harmonia axyridis* on *Spodoptera frugiperda* Eggs

Experimental conditions and replications were as described in Section 2.2.1. The only variable was the number of *S. frugiperda* eggs per treatment, being set at 10, 20, 40, and 80 eggs per dish for both predator treatments.

#### 2.2.3. Effects of Natural Enemy Density on Predatory Response

Ambient conditions for this experiment followed those described above. The number of natural enemies was 1, 2, 3, 4, and 5 larvae per dish and the density of first instar larvae of *S. frugiperda* was 20 per dish. Each treatment was repeated 15 times and the number of larvae remaining in each dish was recorded and analyzed after 24 h.

### 2.3. Statistics and Analysis

The reciprocal conversion of pest density and natural enemy feeding was set to the functional response equation and the theoretical values of each parameter was compared by chi-square test. One-way ANOVA (post hoc Tukey’s HSD test, SPSS 25.0; SPSS, Chicago, IL, USA) (*p* < 0.05) was used to analyze the differences in pest consumption. The homogeneity of variance was tested before analysis with natural logarithmic transformation performed when necessary. The Holling’s Type II Functional Response Model is $N_{a}=\frac{a\prime NT}{1+a\prime T_{h}N}$ [38], where *N_a_* is the net prey consumption rate by the predator during selected time period, *a′* is the instantaneous attack rate, *N* is the density of eggs or larvae, *T* is the predatory time of the predator (1 day) and *T_h_* is the time required to prey on a larva or egg (handling time).

## 3. Results

### 3.1. Predation by Orius sauteri on Larvae and Eggs of Spodoptera frugiperda

**Figure 1:**
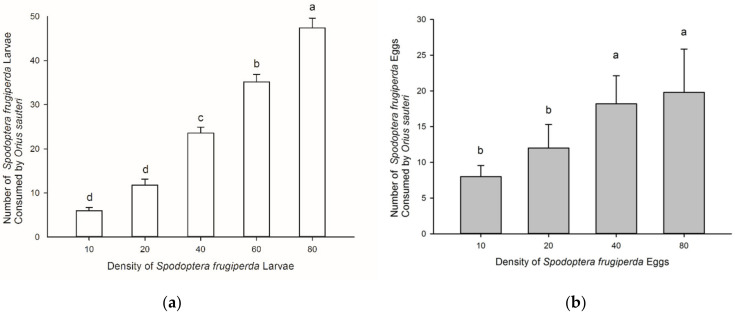
The average predation quantity of 5th instar nymphs of *Orius sauteri* on 1st instar larvae of *Spodoptera frugiperda* at different prey densities (±SE) (**a**) and the number of eggs consumed at different prey densities (±SE) (**b**). Different letters signify significant differences at *p* < 0.05.

The rate of consumption by *O. sauteri* on *S. frugiperda* larvae increased significantly as larval density increased (F = 121.059, df = 4, 70, *p* < 0.05) (Figure 1a). At a larval density of 40/dish, the number of prey consumed was 23.25 ± 1.48 but interestingly when prey availability was reduced (*n* = 10/dish), mean predation rate was only 6 ± 0.71 despite the capacity to consume considerably more prey. Similarly, consumption of *S. frugiperda* eggs increased significantly with higher numbers of available prey (F = 4.691, df = 3, 56, *p* = 0.044) (Figure 1b).

### 3.2. Effect of Predator Density on Consumption of Spodoptera frugiperda by Orius sauteri and Harmonia axyridis

**Figure 2:**
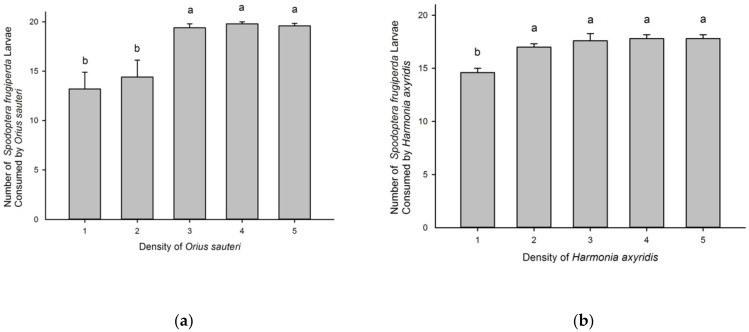
The mean (±SE) number of *Spodoptera frugiperda* 1st instar larvae consumed by *Orius sauteri* (**a**) and *Harmonia axyridis* (**b**) at different predator densities. Different letters signify significant differences at *p* < 0.05.

Increasing the number of predators significantly affected the number of prey consumed by *O. sauteri* (F = 8.492, df = 4, 70, *p* < 0.05) (Figure 2a) and *H. axyridis* (F = 9.24, df = 4, 70, *p* < 0.05) (Figure 2b).

### 3.3. Predation by Harmonia axyridis on Larvae and Eggs of Spodoptera frugiperda

**Figure 3:**
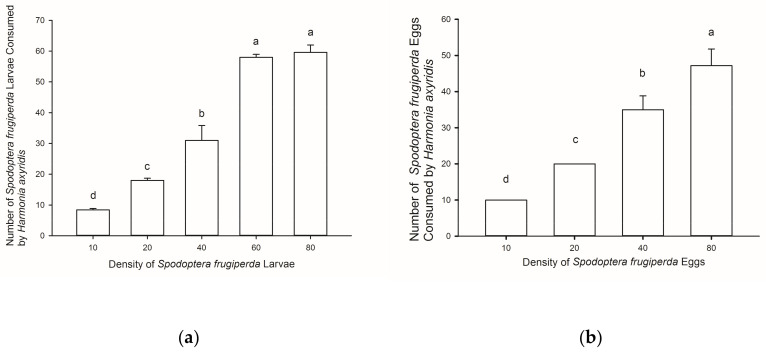
The average predation quantity of adult *Harmonia axyridis* on 1st instar larvae of *Spodoptera frugiperda* at different prey densities (±SE) (**a**) and the number of eggs consumed at different prey densities (±SE) (**b**). Different letters signify significant differences at *p* < 0.05.

The consumption of larvae of *S. frugiperda* by *H. axyridis* increased as the number of available larvae increased (F = 117.319, df = 4, 70, *p* < 0.05). At a larval density of 60/dish, almost all prey were consumed by the single predator (58.00 ± 0.95) and at a lower density (*n* = 10), most prey were eaten (8.4 ± 0.51). The number of eggs of *S. frugiperda* consumed also increased significantly as the number provided increased (F = 30.055, df = 3, 56, *p* < 0.05) (Figure 3b). 

### 3.4. Functional Response of Predators Consuming Spodoptera frugiperda

The consumption of *S. frugiperda* by *O. sauteri* (Table 1) revealed a clear Holling’s Type II functional response (R^2^ > 0.8, *p* > 0.05). The *T_h_* of nymphs of *O. sauteri* on *S. frugiperda* eggs was 0.049 d with a maximum daily feeding rate of 15.19 larvae/d. Although the predation numbers of *O. sauteri* on the pest larvae showed a density dependent manner (Figure 1a), the Type II functional response model revealed that a leveling off tendency would appear when the prey density continuously increase. The *T_h_* of *O. sauteri* on the young larvae of the pest was 0.0008 and the *N_a-max_* was 700.24. 

The functional response of adult *H. axyridis* preying upon *S. frugiperda* (Table 2) also followed a Holling’s Type II functional response (*R^2^* > 0.9, *p* > 0.05). The *T_h_* on *S. frugiperda* eggs was 0.0085 d with a maximum daily feeding rate of 130.73 eggs/d. The *T_h_* of *H.axyridis* on the young larvae of *S. frugiperda* was 0.0006 d and the *N_a-max_* was 1401.1.

## 4. Discussion

The present study examined the predatory capacity of two native predators to eggs and larvae of the invasive lepidopteran herbivore *S. frugiperda*. Results revealed that *H. axyridis* and *O. sauteri* both readily preyed upon eggs and young larvae of *S. frugiperda*, providing a favorable theoretical and practical framework for controlling these pests in the field. Invasive pests often become established in new habitats due to the lack of natural enemies in the new region. Furthermore, given the inherent challenges associated with non-target impacts of introduced exotic generalist predators, evaluation of the suppression capacity of local natural enemies are critical for their successful utilization in biological control programs. During the selection for natural enemies in biological control, the positive density effect of their feeding ability is the basic condition for evaluating their potential role in the field [39]. The Holling’s Type II functional response model was fitted to the predatory effect *H. axyridis* and *O. sauteri* on *S. frugiperda* and our results assist in developing a strategy utilizing different predators in concert with one another for sustainable pest management.

Estimation of the potential for pest suppression through the regression of Holling series modeling has been widely used for selecting optimal candidates in biological control [38,40]. The present study revealed that predation by both predators clearly followed a Holling’s Type II regression and indicated that prey were consumed at increasing rates as density increased. The number of prey consumed by *O. sauteri* on *S. frugiperda* increased as pest density increased, and reached saturation at a high density, indicating that *O. sauteri* could be selected as a potential natural enemy to control *S. frugiperda*. Similar trends were reported on other predator species of *Orius*. Spp, for example, *Orius similis* Zheng (Hemiptera: Anthocoridae) [41], *Orius laevigatus* (Fieber) (Hemiptera: Anthocoridae) [42] and *Orius insidiosus* (Say) (Hemiptera: Anthocoridae) [43]. Similarly, when *H. axyridis* preyed on the eggs or larvae of *S. frugiperda*, they also exhibited a positive density effect. These results indicate the theoretical daily maximum predation quantity of *H. axyridis* and *O. sauteri* on larvae of *S. frugiperda* were 1401.1 and 700.24, respectively, while that on eggs were 130.73 and 15.19, respectively. The duration of consumption (Th) on eggs was previously reported as being shorter than on larvae when *Lycoriella pleuroti* Yang et Zhang was consumed by female *O. sauteri* adults [38]. However, in the present study, the *Th* values from the two natural enemies on eggs were larger than when consuming larvae, which may be related to the physiological structure of *S. frugiperda* eggs. These eggs are surrounded by floc that provides protection for eggs in the natural environment and disrupt predation by natural enemies. *Harmonia axyridis* was reported to show a significant control effect on *Myzus persicae* (Sulzer) [44] and *Aphis gossypii* Glover, also fitting closely to a Holling’s Type II functional response model [45]. Compared with the results presented here, the estimated attack rates on *S. frugiperda* were higher than on aphids, possibly due to the relatively high mobility of *S. frugiperda* larvae and the physical structure of the eggs.

For practical field-based application, once prey density exceeds the threshold density, the natural enemy typically does not increase prey consumption in a given time and will plateau at a theoretical maximum consumption rate. Our estimations infer that *H. axyridis* may consume the eggs and larvae of *S. frugiperda* at a similar level according to the *a′* generated from the fitted formula. However, *O. sauteri* showed higher attacking efficiency to the eggs of *S. frugiperda* but spent more time in handling each egg compared to preying on the larvae. Consistent with the results shown from *H. axyridis*, the physical structure of *S. frugiperda* eggs resulted in a longer handling time and smaller theoretic maximum predation number. In addition to the physical structure of the pest eggs, the predation efficiency was also influenced by temperature [46] and other environmental conditions, but the resistance of host plants was reported to show no effect on the preference of *H. axyridis* [47].

Predators often have a generalist diet, they are sometimes considered ineffective for sustainable management [48]. Our results revealed that *O. sauteri* preferentially consumed 1st instar larvae compared to eggs, and their predation were disturbed by the physical structures of the pests. Parasitoids are often used for the suppression of lepidopterous pests in the field and their releases have been widely studied [11,49]. For example, *Telenomus remus* Nixon (Hymenoptera: Playtgastridae) has already been applied in biological control programs in Africa [50] and could be an effective candidate for biological control. It could, therefore, be speculated that *O. sauteri* could be an effective biological control agent when used in conjunction with parasitoid release. *H. axyridis* may be effective as a supplementary biological control agent and providing longer-term suppression of pest larvae. Pest suppression can be more efficient when a range of different strategies are adopted including the utilization of native natural enemies in conservation biological control. Such approaches can be efficient in the suppression of different pest stages and integrating parasitoid release with underlying populations of generalist predators could result in season-long control of important pests in China.

The present study provides an important baseline of information on the potential management of *S. frugiperda* and the practical application of designing strategies of using *O. sauteri* and *H. axyridis* for sustainable and efficient biological control. However, the results of our tests could not directly predict the predation efficiency in open fields or greenhouses, since the bioassays were conducted in the laboratory. The management of *S. frugiperda* should be developed based on open field experiments considering the utilization of shelter and food resources of non-crop habitats for sustaining natural enemies [51], potentially in combination to other biological control methods (e.g., *Trichogramma* parasitoids [52]).

## Figures and Tables

**Table 1:** Parameters of the functional response of *Orius sauteri* preying on *Spodoptera frugiperda*.

*Orius sauteri*	Prey	Fitted Formula	*R* ^2^	*a′*	*T_h_*	*N_a-max_*	*X*^2^, *p*
Nymph	larvae	*Na* = 0.5626N/(1 − 0.0008N)	0.8591	0.5602	0.0008	700.24	3.333, 1.000
	eggs	*Na* = 0.7444N/(1 + 0.049N)	0.9947	0.7444	0.049	15.19	8.000, 0.924

Note: *N_a-max_* = *a′*/*T_h_* (the theoretical maximum number of prey consumed per day; number); *a′* (per day); *Th* (days).

**Table 2:** Parameters of functional response of *Harmonia axyridis* preying on *Spodoptera frugiperda*.

*Harmonia axyridis*	Prey	Fitted Formula	*R* ^2^	*a′*	*T_h_*	*N_a-max_*	*X*^2^, *p*
Adult	larvae	*Na* = 0.8322N/(1 + 0.0006N)	0.9322	0.8407	0.0006	1401.1	2.400, 1.000
	eggs	*Na* = 1.1112N/(1 + 0.0085N)	0.9654	1.1112	0.0085	130.73	18.250, 0.108

>Note: *N_a-max_* = *a′*/*T_h_* (the theoretical maximum number of prey consumed per day; number); *a′* (per day); *Th* (days).

## Data Availability

The data presented are available in article.
